# Electromyography as a tool to motion analysis for people with Amyotrophic Lateral Sclerosis: A protocol for a systematic review

**DOI:** 10.1371/journal.pone.0302479

**Published:** 2024-05-28

**Authors:** Ana Paula Mendonça Fernandes, Ledycnarf Januário de Holanda, Larissa Coutinho de Lucena, Kelly Evangelista Rodrigues da Silva, Anna Clara Sales Miranda Lopes, Daniel Tezoni Borges, Danilo A. P. Nagem, Ricardo A. de M. Valentim, Laurent Bougrain, Ana Raquel Rodrigues Lindquist

**Affiliations:** 1 Department of Physical Therapy, Federal University of Rio Grande do Norte, Natal, Brazil; 2 Bloorview Research Institute, Holland Bloorview Kids Rehabilitation Hospital, Toronto, Canada; 3 Laboratory of Technological Innovation in Health, Federal University of Rio Grande do Norte, Natal, Brazil; 4 Department of Biomedical Engineering, Federal University of Rio Grande do Norte, Natal, Brazil; 5 Dept. of complex system, Artificial intelligence and robotics at Loria, University of Lorraine, Nancy, France; Nnamdi Azikiwe University, NIGERIA

## Abstract

Biomechanical analysis of human movement plays an essential role in understanding functional changes in people with Amyotrophic Lateral Sclerosis (ALS), providing information on muscle impairment. Studies suggest that surface electromyography (sEMG) may be able to quantify muscle activity, identify levels of fatigue, assess muscle strength, and monitor variation in limb movement. In this article, a systematic review protocol will analyze the psychometric properties of the sEMG regarding the clinical data on the skeletal muscles of people with ALS. This protocol uses the Preferred Reporting Items for Systematic Reviews and Meta-Analyses (PRISMA) methodological tool. A specific field structure was defined to reach each phase. Nine scientific databases (PubMed, Web of Science, Embase, Elsevier, IEEE, Google Scholar, SciELO, PEDro, LILACS E CENTRAL) were searched. The framework developed will extract data (i.e. study information, sample information, sEMG information, intervention, and outcomes) from the selected studies using a rigorous approach. The data will be described quantitatively using frequency and trend analysis methods, and heterogeneity between the included studies will be assessed using the I2 test. The risk of bias will be summarized using the most recent prediction model risk of bias assessment tool. Be sure to include relevant statistics here, such as sample sizes, response rates, P values or Confidence Intervals. Be specific (by stating the value) rather than general (eg, “there were differences between the groups”). This protocol will map out the construction of a systematic review that will identify and synthesize the advances in movement analysis of people with ALS through sEMG, using data extracted from articles.

## Introduction

The ability to capture electrical nerve signals from the motor units of skeletal muscles, controlled by motor neurons, is provided by surface electromyography (sEMG) [1–3]. By detecting information on the signal frequency, in a range between 0 and 500 Hz, and the amplitude of muscle activation, between 0.01 mV and 10 mV, using invasive (intramuscular EMG) and non-invasive (surface EMG) electrodes, it is possible to measure bioelectric changes in the skeletal muscles of people with neurological diseases, including stroke, Parkinson’s disease and peripheral neuropathy [1–5].

Among the conditions with neuromuscular involvement, Amyotrophic Lateral Sclerosis (ALS) stands out, defined as a degenerative disease of the upper motor neurons (UMN) and lower motor neurons (LMN) of the central and peripheral nervous system, located in the spinal cord, brainstem and cortex [6–8]. Initially, ALS of spinal origin presents a clinical picture with physical and functional alterations in the upper and lower limbs, characterized by movement restrictions, weakness, fasciculations, atrophy, and pain, while bulbar ALS is characterized by dysarthria, dysphagia, and sialorrhea [6].

In the context of ALS, sEMG can detect abnormalities in the motor unit (MU), concerning the firing rate, recruitment threshold, and quantity of MU, as well as providing structural information about the muscle innervation zone [9]. Thus, it is possible to identify fasciculations, which are an important symptom for the prognosis of lower motor neuron dysfunction, the level of fatigue and muscle strength, as well as signs of reinnervation and denervation [10–12]. Nishikawa et al. [9] identified through a High-Density EMG that people with ALS have significantly higher MU firing rates than healthy individuals, with a direct correlation with the severity of the disease. Furthermore, an increased excitability in recruitment was identified, in parallel with a compensatory increase in MU activity, as a way of compensating for the reduction in the quantity of MU.

From the perspective involving the assessment of fasciculations, Jing Ma et al. [12] demonstrated using needle electromyography that the detection rates of fasciculation and the number of muscles with fasciculation in patients with ALS were significantly higher than those in healthy individuals. Thus, a continuous and multifocal pattern of fasciculations was observed in ALS patients compared to healthy patients. Also, muscle fatigue was observed by Goncalves et al. [13], in which the group of patients with ALS showed a decrease in the median frequency of the sEMG signal of the masseter and temporal muscles compared to the group of healthy patients.

In addition to the discussed functions of electromyography, its coupling with inertial meters, such as the accelerometer (ACC), allows body movement to be analyzed [14]. In this context, Holanda et al. used surface electromyography in ALS patients, with a sampling frequency of 1926 Hz and 1111 Hz for their electromyography sensors and 148 Hz in relation to the ACC, and showed that the amplitude of the ACC was significantly lower in ALS patients than in healthy patients [15].

Thus, UM-related data can provide an important physiological index for understanding the pathophysiology of ALS, indicating the usefulness of sEMG as a method for non-invasive assessment of neuromuscular degeneration [9]. Furthermore, it can serve as an additional diagnostic tool, given that there is variability in initial symptoms, associated with a lack of specific biomarkers and difficulty in diagnosing at the beginning of the disease, thus prolonging the time to diagnosis [16, 17].

Bashford and cols [11] in a systematic review, identified a wide variety of analytical techniques involving decomposition and estimation of motor unit numbers and measures of neuronal hyperexcitability or neuromuscular architecture, with sEMG being a technique that offers significant practical and analytical flexibility compared to invasive techniques.

This way, by analyzing body movement data acquired through EMGs in individu- als with ALS, healthcare professionals can provide assistance based on objective and precise information rather than solely relying on subjective assessments [4, 17, 18]. This analysis is pivotal for public health, considering the uniqueness of each patient and the varied progression of ALS, necessitating individualized care. Through a comprehensive analysis of all variables, the treatment plan devised following assess- ment of each patient’s data would not only prevent underestimating training but also avoid exacerbating the limits of muscle fatigue, a clinical indicator that impairs quality of life and leads to disability in individuals with ALS [11, 13, 19].

This review aims to identify the clinical data that can be extracted using sEMG in order to corroborate the analysis of movement in people with ALS. We will seek to identify clinical motor parameters and muscle groups involved in the analysis, as well as examine the various forms of assessment and/or interventions that use sEMG as a tool. In addition, we will discuss the physical characteristics of the sEMG devices used and describe the procedures adopted for analyzing the data obtained.

This systematic review protocol will examine sEMG data as parameters for investigating skeletal muscle movement in people with ALS. The data collection will allow us to establish correlations between published studies, consolidating evidence that will improve our understanding of the usefulness of sEMG in assessing muscle function in ALS patients. We hope that the results obtained will provide valuable insights to design more effective therapeutic approaches for this neuromuscular condition and that we can make a significant contribution to the scientific literature and provide further innovation in clinical care for this disease.

## Methods

### Study design

This protocol is based on the Preferred Reporting Items for Systematic Reviews and Meta-Analyses (PRISMA) methodology tool S2 Appendix, will utilize the flowchart according to PRISMA S3 Appendix and has been registered in the International Prospective Register of Systematic Reviews (PROSPERO) with ID CRD42023465596 [20] The evaluation will start in July 2024 and end in February 2026. Thus, the initial exploration will include studies and aspects related to the description of the sEMG associated or not with validated scales for people with ALS or for specific outcomes of the disease, processing techniques, description of the intervention and analysis, characteristics of the participants, and clinical data evaluated.

### Search strategy and eligibility criteria

The eligibility criteria were established according to the PICOTS strategy (Participants, Interventions, Comparisons, Outcomes, Time frame, and Study design) [21]. The search strategy will be adapted to the reality of each database established for research. This review will explore specific issues such as the characteristics and feasibility of sEMG in identifying clinical motor symptoms in people with ALS. Therefore, the review questions were previously defined to provide the roadmap for the subsequent steps. Thus, a set of questions was drawn up for each of the following aspects: (1) sEMG characteristics, (2) processing techniques, and (3) clinical data analyzed. It is worth noting that the research questions in this study will not be limited to those presented in Table 1. Other questions may be discussed based on the analysis of the data in the preparation of the systematic review.

**Table 1:** Research questions.

Aspects	List of Questions
Identified clinical data	What motor clinical parameters, such as fatigue, fasciculation, paresis/plegia, can be evaluated using sEMG in individuals diagnosed with ALS?
Analyzed musculature	Which muscle groups are assessed via sEMG? Does this encompass upper limbs, lower limbs, trunk, cervical region, head, and neck?
Assessment	How was the assessment conducted using sEMG? What activity was employed, the duration of data collection, and the rest period?
	Were tools, such as scales, implemented to evaluate individuals diagnosed with ALS?
	What is the patient classification based on clinical data, scale scores, time interval between symptom onset and diagnosis, and duration since diagnosis?
sEMG associated with intervention	Was sEMG utilized as biofeedback during interventions in ALS individuals?
Properties of EMGs	What are the physical properties of sEMG employed in the analyses, such as electrode type, sampling frequency, and portability?
Signal analysis	How was the sEMG signal analysis performed regarding calibration, signal acquisition and registration, amplification, normalization, and signal processing?

### Types of participants

Studies will be considered with humans who have a clinical diagnosis of ALS confirmed by a neurologist’s report, according to the El Escorial revisited [22] criteria, not associated with other neurological conditions.

### Types of intervention

We will consider studies that have evaluated people with ALS with sEMG, using surface electromyography associated or not with validated scales for ALS.

### Types of comparison

We will include studies comparing the impacts of sEMG in relation to the time domain, the frequency domain, or the combination of time and frequency. We will also compare the characteristics of the sEMG devices used.

### Types of devices

The different types of sEMG devices will be considered, analyzing their physical characteristics such as portability, use of wires, type of electrode, and sampling frequency, as well as whether or not they integrate inertial sensors.

### Types of outcome measures

Studies that address the functional kinetic assessment of ALS patients in skeletal musculature will be included, excluding studies that only assess musculature and/or respiratory, cognitive, and/or bulbar function.

### Time considered of measurement

The outcome measures will be considered at the following evaluation moments:

1. Before the intervention: the last measurement before the intervention (base- line);
2. After the intervention (short-term): the first measurement after the end of the intervention (post-training);
3. After the intervention (medium or long term): the second measurement after the end of the intervention (follow-up).

### Type of studies

Observational cross-sectional studies, longitudinal studies, quasi-experimental studies, experimental studies, and nonrandomized and randomized clinical trials will be considered, and review studies, book chapters, and duplicate articles will be excluded.

## Search methods for identifying studies

### Search strategy

The search strategy will use keywords and synonyms based on the Medical Sub- ject Headings (MeSH) and Health Sciences Descriptors (Decs). The descriptors are broad, covering the body segment, physical rehabilitation, clinical data, electromyo- graphy, and the sample of interest, as shown in Table 2. Our team, composed of researchers from different areas of interest, devised a list of relevant terms for the databases, presented in Supporting information S1 Appendix.

**Table 2:** Main concepts and related keywords.

Concept	Matching Keywords
Body segment	Upper Extremity, Upper Limb, trunk musculature, cervical, Lower extremity, Lower limb, Hand, Elbow, Wrist, Shoulder, Forearm, Arm, Fingers, Foot, Ankle, Knee, Hip, Fingers
Physical Rehabilitation	Physical Rehabilitation, Motor Rehabilitation, Physical Medicine, Telerehabilitation, Physiotherapy, Functional Result
Clinical data	Motor signs and symptoms, fasciculations, cramps, weakness, fatigue, stiffness, spasticity, clonus, hypertonia, hyperreflexia
Eletromyography	sEMG, EMG, MUSCLE ACTIVITY, MUSCLE ACTIVATION
Mechanomyography	MMG
Amyotrophic Lateral Sclerosis	ALS—Amyotrophic Lateral Sclerosis ALS Amyotrophic Lateral Sclerosis Amyotrophic Lateral Sclerosis Parkinsonism Dementia Complex 1 Amyotrophic Lateral Sclerosis With Dementia Amyotrophic Lateral Sclerosis, Guam Form Amyotrophic Lateral Sclerosis, Parkinsonism Dementia Complex of Guam Amyotrophic Lateral Sclerosis, Parkinsonism-Dementia Complex of Guam Amyotrophic Lateral Sclerosis-Parkinsonism-Dementia Complex 1 Charcot Disease Dementia With Amyotrophic Lateral Sclerosis Disease, Guam Disease, Lou-Gehrigs Gehrig Disease Gehrig’s Disease Gehrigs Disease Guam Disease Guam Form of Amyotrophic Lateral Sclerosis Lou Gehrig Disease Lou Gehrig’s Disease Lou-Gehrigs Disease Motor Neuron Disease, Amyotrophic Lateral Sclerosis Sclerosis, Amyotrophic Lateral

We plan to conduct a sensitive and non-specific search of the literature. Thus, the search terms will be kept broad, and irrelevant studies will be eliminated in the study selection phase. All article searches will be carried out without restriction as to language or year of publication.

### Electronic searches

Articles will be selected, without restrictions on language or publication date, from electronic databases, reference lists of relevant literature, of important journals, and conference proceedings presenting relevant publications on the review topic. Numerous keywords will be combined to formulate the search strings. From this perspective, the database search will be divided into several stages:

1. A comprehensive search will be carried out in the following databases: MED- LINE (PubMed), Web of Science (Clarivate Analytics), Embase (Elsevier), IEEE Xplore Digital Library (IEEE), Scopus (Elsevier), Google Scholar (Google), Scientific Electronic Library Online (SciELO), Physiotherapy Ev- idence Database (PEDro), Cochrane Central Register of Controlled Trials (CENTRAL) and Latin American and Caribbean Health Science Information database (LILACS).
2. In addition, ongoing and/or unpublished clinical trials will be searched in the ClinialTrials.gov (clinicaltrials.gov) database and in the World Health Organi zation’s International Clinical Trials Registry Platform (www.who.int/ictrp/en/)
3. Grey literature: The reference list of all included studies will be checked man- ually to look for additional relevant.

## Data collection and analysis

### Search selection

The studies will be selected initially from the titles and abstracts and, in case of doubt, from the full articles obtained in our search activities, studies that did not meet the requirements of the review protocol in terms of type of study, partici- pants, intervention and/or comparison group, time frame, and study design will be excluded. Based on the inclusion criteria described above, three independent reviewers (APMF, KERS, ACSML) will screen the titles and abstracts identified during the electronic and manual searches to determine their eligibility. Any disagreements will be resolved with a fourth reviewer (LJH). The information from the selected stud- ies will be imported into the Rayyan systematic review software [23] to help exclude studies that do not meet the inclusion criteria and duplicate studies. If the title or abstract does not provide sufficient information for inclusion, the full text will be obtained for a complete review.

### Extraction and data management

The included studies will proceed to data extraction and quality assessment according to the stages of the review. Five independent reviewers (APMF, KERS, ACSML, DTB, LCL) will extract the outcome data from the included studies. Any disagreements that arise will be resolved initially by discussion between the reviewers, or, if necessary, with the help of a sixth reviewer (LJH). A table will be created for data extraction, divided into: (1) study information, including the year of publication, author information, funding or sponsorship infor- mation, type of study, and journal name; (2) sample information, including popu- lation, sample size, diagnosis time, associated pathologies, clinical data, validated scales; (3) information about the sEMG, such as sampling frequency, portability, wired or wireless, inertial sensors included, signal analysis methods; (4) interven- tion, such as type of intervention, frequency, duration, and positioning of the sensor, description of the task, use of conductive gel/adhesive; (5) results.

### Missing data

Regarding the missing data, the researchers will contact the authors of the respective studies to request missing information.

## Data analysis

### Methodological quality

The GRADE system (Grading of Recommendations Assessment, Development, and Evaluation) [24] will be used to grade the quality of the evidence, using each outcome of the studies analyzed. It is classified into four levels: high, moderate, low, and very low. Taking into account study design, methodological limitations (risk of bias), inconsistency, indirect evidence, imprecision, publication bias, magnitude of effect, dose-response gradient, and residual confounding factors [25].

### Risk of bias

The pre-selected articles will be assessed for methodological quality using the Pre- diction model Risk Of Bias Assessment Tool (PROBAST), which includes 20 ques- tions divided into four domains (participants, predictors, outcome, and analysis), and the risk of bias is judged as low, high, or unclear [26].

### Sensitivity analysis and heterogeneity

Heterogeneity between studies will be assessed using I2 [27] and interpreted as “may not be important” (0–40%), “may represent moderate heterogeneity” (30–60%), “may represent substantial heterogeneity” (50–90%) and “considerable heterogeneity” (75–100%) [19]. Sensitivity analyses will be carried out when missing data suggests an important bias. Studies with a high risk of bias (i.e. allocation and assessment of unblinded outcomes) will be excluded. For homogeneous studies, a meta-analysis will be carried out with the appropriate data, generating a synthesis of previous research, and quantitative conclusions.

## Discussion

This systematic review protocol represents a significant milestone in the field of analyzing the movement of people with ALS using sEMG data, as it is the first to synthesize existing evidence. The main objective is to map and synthesize advances in this area through a detailed systematic review protocol, using the framework proposed by PRISMA.

Clinical data on the skeletal musculature of people with ALS analyzed using sEMG, correlated or not with functional scales, will be summarized. In addition, studies using this equipment as a means of biofeedback will be included. This way, we will map the clinical relevance of sEMG in this population.

Furthermore, the analysis of quantitative sEMG data can be highly informative, particularly when employing features extracted from temporal, frequency, and time-frequency domains, as well as the joint analysis of EMG spectra and amplitudes [28–30]. Each of these methods can be linked to ALS symptoms, providing means to extract characteristics and enhance the assessment of movement in individuals affected by this health condition. This encompasses the analysis of symptoms such as fatigue, fasciculations, initial muscle activation, and its intensity, as well as variations in upper limb movement. These pieces of information hold great importance as they can significantly contribute to ALS electrodiagnosis, potentially reducing public expenditures. Additionally, these methods can be employed in prescribing individualized therapeutic approaches, monitoring disease progression, and providing insights for assistive technology control [31, 32].

It is hoped that future systematic reviews will adopt our protocol as an improved version of previous frameworks, providing more detail on the signs and symptoms extracted, data analysis, categorization, and technical analyses. This more comprehensive and detailed approach will contribute to the advancement of knowledge in this area and to a better understanding of the psychometric properties of the sEMG in people with ALS.

## Supporting information

S1 Appendix We improved the catalog of significant terms for the review, customized to fit the different databases. (PDF)

S2 Appendix PRISMA-P 2015 checklist. (PDF)

S3 Appendix Systematic review, which will be initiated after the publication of the protocol, will utilize the flowchart according to PRISMA guidelines. (PDF)
