# Supplementary material for: Electromyography as a tool to motion analysis for people with Amyotrophic Lateral Sclerosis: A protocol for a systematic review
Source: PLoS One. 2024 May 28;19(5):e0302479. doi: 10.1371/journal.pone.0302479 (PMC11132455; doi:10.1371/journal.pone.0302479)
Supplement: S1 Appendix — (PDF) [file pone.0302479.s001.pdf]

S1 APPENDIX - DATABASE SEARCH STRATEGY

- MEDLINE (PubMed)

((Upper Extremity) OR (Upper Limb)) OR (Hand)) OR (Elbow)) OR (Wrist)) OR (Shoulder)) OR (Forearm)) OR (Arm)) OR (Fingers)) OR (trunk musculature)) OR (cervical trunk musculature)) OR (Lower extremity)) OR (Lower limb)) OR (Foot)) OR (Ankle)) OR (Knee)) OR (Hip)) OR (Fingers)) AND (Physical Rehabilitation)) OR (Motor Rehabilitation)) OR (Physical Medicine)) OR (Telerehabilitation)) OR (Physiotherapy)) OR (Functional Result)) AND (Motor signs)) OR (motor symptoms)) OR (fasciculations)) OR (cramps)) OR (weakness)) OR (fatigue)) OR (stiffness)) OR (spasticity)) OR (clonus)) OR (hypertonia)) OR (hyperreflexia)) AND (sEMG)) OR (EMG)) OR (MUSCLE ACTIVITY)) OR (MUSCLE ACTIVATION)) OR (Electromyographies)) OR (Surface Electromyography)) OR (Electromyographies, Surface)) OR (Electromyography, Surface)) OR (Surface Electromyographies)) OR (Electromyogram)) OR (Electromyograms)) AND (ALS)) OR (ALS - Amyotrophic Lateral Sclerosis)) OR (ALS Amyotrophic Lateral Sclerosis)) OR (Amyotrophic Lateral Sclerosis Parkinsonism Dementia Complex 1)) OR (Amyotrophic Lateral Sclerosis With Dementia)) OR (Amyotrophic Lateral Sclerosis, Guam Form)) OR (Amyotrophic Lateral Sclerosis, Parkinsonism Dementia Complex of Guam)) OR (Amyotrophic Lateral Sclerosis, Parkinsonism-Dementia Complex of Guam)) OR (Amyotrophic Lateral Sclerosis-Parkinsonism-Dementia Complex 1)) OR (Charcot Disease)) OR (Dementia With Amyotrophic Lateral Sclerosis)) OR (Disease, Guam)) OR (Disease, Lou-Gehrigs)) OR (Gehrig Disease)) OR (Gehrig's Disease)) OR (Gehrigs Disease)) OR (Guam Disease)) OR (Guam Form of Amyotrophic Lateral Sclerosis)) OR (Lou Gehrig Disease)) OR (Lou Gehrig's Disease)) OR (Lou-Gehrigs Disease)) OR (Motor Neuron Disease, Amyotrophic Lateral Sclerosis)) OR (Sclerosis, Amyotrophic Lateral)

- Web of Science (Clarivate Analytics)

AAL = (“Upper Extremity” OR “Upper Limb” OR “Hand” OR “Elbow” OR “Wrist” OR “Shoulder” OR “Forearm” OR “Arm” OR “Fingers” OR “trunk musculature” OR “cervical trunk musculature” OR “Lower extremity” OR “Lower limb” OR “Foot” OR “Ankle” OR “Knee” OR “Hip” OR “Fingers”) AND (“Physical Rehabilitation” OR “Motor Rehabilitation” OR “Physical Medicine” OR “Telerehabilitation” OR “Physiotherapy” OR “Functional Result”) AND (“Motor signs” OR “motor symptoms” OR “fasciculations” OR “cramps” OR “weakness” OR “fatigue” OR “stiffness” OR “spasticity” OR “clonus” OR “hypertonia” OR “hyperreflexia”) AND (“sEMG” OR “EMG” OR “MUSCLE ACTIVITY” OR “MUSCLE ACTIVATION” OR “Electromyographies” OR “Surface Electromyography” OR “Electromyographies, Surface” OR “Electromyography, Surface” OR “Surface Electromyographies” OR “Electromyogram” OR “Electromyograms”) AND (“ALS” OR “ALS - Amyotrophic Lateral Sclerosis” OR “ALS Amyotrophic Lateral Sclerosis” OR “Amyotrophic Lateral Sclerosis Parkinsonism Dementia Complex 1” OR “Amyotrophic Lateral Sclerosis With Dementia” OR “Amyotrophic Lateral Sclerosis, Guam Form” OR “Amyotrophic Lateral Sclerosis, Parkinsonism Dementia Complex of Guam” OR “Amyotrophic Lateral Sclerosis, Parkinsonism-Dementia Complex of Guam” OR

“Amyotrophic Lateral Sclerosis-Parkinsonism-Dementia Complex 1” OR “Charcot Disease” OR “Dementia With Amyotrophic Lateral Sclerosis” OR “Disease, Guam” OR “Disease, Lou-Gehrigs” OR “Gehrig Disease” OR “Gehrig's Disease” OR “Gehrigs Disease” OR “Guam Disease” OR “Guam Form of Amyotrophic Lateral Sclerosis” OR “Lou Gehrig Disease” OR “Lou Gehrig's Disease” OR “Lou-Gehrigs Disease” OR “Motor Neuron Disease, Amyotrophic Lateral Sclerosis” OR “Sclerosis, Amyotrophic Lateral”)

- Embase (Elsevier)

(‘Upper Extremity’ OR ‘Upper Limb’ OR ‘Hand’ OR ‘Elbow’ OR ‘Wrist’ OR ‘Shoulder’ OR ‘Forearm’ OR ‘Arm’ OR ‘Fingers’ OR ‘trunk musculature’ OR ‘cervical trunk musculature’ OR ‘Lower extremity’ OR ‘Lower limb’ OR ‘Foot’ OR ‘Ankle’ OR ‘Knee’ OR ‘Hip’ OR ‘Fingers’) AND (‘Physical Rehabilitation’ OR ‘Motor Rehabilitation’ OR ‘Physical Medicine’ OR ‘Telerehabilitation’ OR ‘Physiotherapy’ OR ‘Functional Result’) AND (‘Motor signs’ OR ‘motor symptoms’ OR ‘fasciculations’ OR ‘cramps’ OR ‘weakness’ OR ‘fatigue’ OR ‘stiffness’ OR ‘spasticity’ OR ‘clonus’ OR ‘hypertonia’ OR ‘hyperreflexia’) AND (‘sEMG’ OR ‘EMG’ OR ‘MUSCLE ACTIVITY’ OR ‘MUSCLE ACTIVATION’ OR ‘Electromyographies’ OR ‘Surface Electromyography’ OR ‘Electromyographies, Surface’ OR ‘Electromyography, Surface’ OR ‘Surface Electromyographies’ OR ‘Electromyogram’ OR ‘Electromyograms’) AND (‘ALS’ OR ‘ALS - Amyotrophic Lateral Sclerosis’ OR ‘ALS Amyotrophic Lateral Sclerosis’ OR ‘Amyotrophic Lateral Sclerosis Parkinsonism Dementia Complex 1’ OR ‘Amyotrophic Lateral Sclerosis With Dementia’ OR ‘Amyotrophic Lateral Sclerosis, Guam Form’ OR ‘Amyotrophic Lateral Sclerosis, Parkinsonism Dementia Complex of Guam’ OR ‘Amyotrophic Lateral Sclerosis, Parkinsonism-Dementia Complex of Guam’ OR ‘Amyotrophic Lateral Sclerosis-Parkinsonism-Dementia Complex 1’ OR ‘Charcot Disease’ OR ‘Dementia With Amyotrophic Lateral Sclerosis’ OR ‘Disease, Guam’ OR ‘Disease, Lou-Gehrigs’ OR ‘Gehrig Disease’ OR ‘Gehrig's Disease’ OR ‘Gehrigs Disease’ OR ‘Guam Disease’ OR ‘Guam Form of Amyotrophic Lateral Sclerosis’ OR ‘Lou Gehrig Disease’ OR ‘Lou Gehrig's Disease’ OR ‘Lou-Gehrigs Disease’ OR ‘Motor Neuron Disease, Amyotrophic Lateral Sclerosis’ OR ‘Sclerosis, Amyotrophic Lateral’)

- IEEE Xplore Digital Library (IEEE)

(((Upper Extremity) OR (Upper Limb)) OR (Hand)) OR (Elbow)) OR (Wrist)) OR (Shoulder)) OR (Forearm)) OR (Arm)) OR (Fingers)) OR (trunk musculature)) OR (cervical trunk musculature)) OR (Lower extremity)) OR (Lower limb)) OR (Foot)) OR (Ankle)) OR (Knee)) OR (Hip)) OR (Fingers)) AND (Physical Rehabilitation)) OR (Motor Rehabilitation)) OR (Physical Medicine)) OR (Telerehabilitation)) OR (Physiotherapy)) OR (Functional Result)) AND (Motor signs)) OR (motor symptoms)) OR (fasciculations)) OR (cramps)) OR (weakness)) OR (fatigue)) OR (stiffness)) OR (spasticity)) OR (clonus)) OR (hypertonia)) OR (hyperreflexia)) AND (sEMG)) OR (EMG)) OR (MUSCLE ACTIVITY)) OR (MUSCLE ACTIVATION)) OR (Electromyographies)) OR (Surface Electromyography)) OR (Electromyographies, Surface)) OR (Electromyography, Surface)) OR (Surface Electromyographies)) OR (Electromyogram)) OR (Electromyograms)) AND (ALS)) OR (ALS - Amyotrophic Lateral Sclerosis)) OR (ALS

Amyotrophic Lateral Sclerosis)) OR (Amyotrophic Lateral Sclerosis Parkinsonism Dementia Complex 1)) OR (Amyotrophic Lateral Sclerosis With Dementia)) OR (Amyotrophic Lateral Sclerosis, Guam Form)) OR (Amyotrophic Lateral Sclerosis, Parkinsonism Dementia Complex of Guam)) OR (Amyotrophic Lateral Sclerosis, Parkinsonism-Dementia Complex of Guam)) OR (Amyotrophic Lateral Sclerosis-Parkinsonism-Dementia Complex 1)) OR (Charcot Disease)) OR (Dementia With Amyotrophic Lateral Sclerosis)) OR (Disease, Guam)) OR (Disease, Lou-Gehrigs)) OR (Gehrig Disease)) OR (Gehrig's Disease)) OR (Gehrigs Disease)) OR (Guam Disease)) OR (Guam Form of Amyotrophic Lateral Sclerosis)) OR (Lou Gehrig Disease)) OR (Lou Gehrig's Disease)) OR (Lou-Gehrigs Disease)) OR (Motor Neuron Disease, Amyotrophic Lateral Sclerosis)) OR (Sclerosis, Amyotrophic Lateral)

- Scopus (Elsevier)

(‘Upper Extremity’ OR ‘Upper Limb’ OR ‘Hand’ OR ‘Elbow’ OR ‘Wrist’ OR ‘Shoulder’ OR ‘Forearm’ OR ‘Arm’ OR ‘Fingers’ OR ‘trunk musculature’ OR ‘cervical trunk musculature’ OR ‘Lower extremity’ OR ‘Lower limb’ OR ‘Foot’ OR ‘Ankle’ OR ‘Knee’ OR ‘Hip’ OR ‘Fingers’) AND (‘Physical Rehabilitation’ OR ‘Motor Rehabilitation’ OR ‘Physical Medicine’ OR ‘Telerehabilitation’ OR ‘Physiotherapy’ OR ‘Functional Result’) AND (‘Motor signs’ OR ‘motor symptoms’ OR ‘fasciculations’ OR ‘cramps’ OR ‘weakness’ OR ‘fatigue’ OR ‘stiffness’ OR ‘spasticity’ OR ‘clonus’ OR ‘hypertonia’ OR ‘hyperreflexia’) AND (‘sEMG’ OR ‘EMG’ OR ‘MUSCLE ACTIVITY’ OR ‘MUSCLE ACTIVATION’ OR ‘Electromyographies’ OR ‘Surface Electromyography’ OR ‘Electromyographies, Surface’ OR ‘Electromyography, Surface’ OR ‘Surface Electromyographies’ OR ‘Electromyogram’ OR ‘Electromyograms’) AND (‘ALS’ OR ‘ALS - Amyotrophic Lateral Sclerosis’ OR ‘ALS Amyotrophic Lateral Sclerosis’ OR ‘Amyotrophic Lateral Sclerosis Parkinsonism Dementia Complex 1’ OR ‘Amyotrophic Lateral Sclerosis With Dementia’ OR ‘Amyotrophic Lateral Sclerosis, Guam Form’ OR ‘Amyotrophic Lateral Sclerosis, Parkinsonism Dementia Complex of Guam’ OR ‘Amyotrophic Lateral Sclerosis, Parkinsonism-Dementia Complex of Guam’ OR ‘Amyotrophic Lateral Sclerosis-Parkinsonism-Dementia Complex 1’ OR ‘Charcot Disease’ OR ‘Dementia With Amyotrophic Lateral Sclerosis’ OR ‘Disease, Guam’ OR ‘Disease, Lou-Gehrigs’ OR ‘Gehrig Disease’ OR ‘Gehrig's Disease’ OR ‘Gehrigs Disease’ OR ‘Guam Disease’ OR ‘Guam Form of Amyotrophic Lateral Sclerosis’ OR ‘Lou Gehrig Disease’ OR ‘Lou Gehrig's Disease’ OR ‘Lou-Gehrigs Disease’ OR ‘Motor Neuron Disease, Amyotrophic Lateral Sclerosis’ OR ‘Sclerosis, Amyotrophic Lateral’)

- Google Scholar (Google)

(“Upper Extremity” OR “Upper Limb” OR “Hand” OR “Elbow” OR “Wrist” OR “Shoulder” OR “Forearm” OR “Arm” OR “Fingers” OR “trunk musculature” OR “cervical trunk musculature” OR “Lower extremity” OR “Lower limb” OR “Foot” OR “Ankle” OR “Knee” OR “Hip” OR “Fingers”) AND (“Physical Rehabilitation” OR “Motor Rehabilitation” OR “Physical Medicine” OR “Telerehabilitation” OR “Physiotherapy” OR “Functional Result”) AND (“Motor signs” OR “motor symptoms” OR “fasciculations” OR “cramps” OR

“weakness” OR “fatigue” OR “stiffness” OR “spasticity” OR “clonus” OR “hypertonia” OR “hyperreflexia”) AND (“sEMG” OR “EMG” OR “MUSCLE ACTIVITY” OR “MUSCLE ACTIVATION” OR “Electromyographies” OR “Surface Electromyography” OR “Electromyographies, Surface” OR “Electromyography, Surface” OR “Surface Electromyographies” OR “Electromyogram” OR “Electromyograms”) AND (“ALS” OR “ALS - Amyotrophic Lateral Sclerosis” OR “ALS Amyotrophic Lateral Sclerosis” OR “Amyotrophic Lateral Sclerosis Parkinsonism Dementia Complex 1” OR “Amyotrophic Lateral Sclerosis With Dementia” OR “Amyotrophic Lateral Sclerosis, Guam Form” OR “Amyotrophic Lateral Sclerosis, Parkinsonism Dementia Complex of Guam” OR “Amyotrophic Lateral Sclerosis, Parkinsonism-Dementia Complex of Guam” OR “Amyotrophic Lateral Sclerosis-Parkinsonism-Dementia Complex 1” OR “Charcot Disease” OR “Dementia With Amyotrophic Lateral Sclerosis” OR “Disease, Guam” OR “Disease, Lou-Gehrigs” OR “Gehrig Disease” OR “Gehrig's Disease” OR “Gehrigs Disease” OR “Guam Disease” OR “Guam Form of Amyotrophic Lateral Sclerosis” OR “Lou Gehrig Disease” OR “Lou Gehrig's Disease” OR “Lou-Gehrigs Disease” OR “Motor Neuron Disease, Amyotrophic Lateral Sclerosis” OR “Sclerosis, Amyotrophic Lateral”)

- Scientific Electronic Library Online (SciELO)

((Upper Extremity) OR (Upper Limb) OR (Hand) OR (Elbow) OR (Wrist) OR (Shoulder) OR (Forearm) OR (Arm)) OR (Fingers) OR (trunk musculature) OR (cervical trunk musculature) OR (Lower extremity) OR (Lower limb) OR (Foot) OR (Ankle) OR (Knee) OR (Hip) OR (Fingers)) AND ((Physical Rehabilitation) OR (Motor Rehabilitation) OR (Physical Medicine) OR (Telerehabilitation) OR (Physiotherapy) OR (Functional Result)) AND ((Motor signs) OR (motor symptoms) OR (fasciculations) OR (cramps) OR (weakness) OR (fatigue) OR (stiffness) OR (spasticity) OR (clonus) OR (hypertonia) OR (hyperreflexia)) AND ((sEMG) OR (EMG) OR (MUSCLE ACTIVITY) OR (MUSCLE ACTIVATION) OR (Electromyographies) OR (Surface Electromyography) OR (Electromyographies, Surface) OR (Electromyography, Surface) OR (Surface Electromyographies) OR (Electromyogram) OR (Electromyograms)) AND ((ALS) OR (ALS - Amyotrophic Lateral Sclerosis) OR (ALS Amyotrophic Lateral Sclerosis) OR (Amyotrophic Lateral Sclerosis Parkinsonism Dementia Complex 1) OR (Amyotrophic Lateral Sclerosis With Dementia) OR (Amyotrophic Lateral Sclerosis, Guam Form) OR (Amyotrophic Lateral Sclerosis, Parkinsonism Dementia Complex of Guam) OR (Amyotrophic Lateral Sclerosis, Parkinsonism-Dementia Complex of Guam) OR (Amyotrophic Lateral Sclerosis-Parkinsonism-Dementia Complex 1) OR (Charcot Disease) OR (Dementia With Amyotrophic Lateral Sclerosis) OR (Disease, Guam) OR (Disease, Lou-Gehrigs) OR (Gehrig Disease) OR (Gehrig's Disease) OR (Gehrigs Disease) OR (Guam Disease) OR (Guam Form of Amyotrophic Lateral Sclerosis) OR (Lou Gehrig Disease) OR (Lou Gehrig's Disease) OR (Lou-Gehrigs Disease) OR (Motor Neuron Disease, Amyotrophic Lateral Sclerosis) OR (Sclerosis, Amyotrophic Lateral))

- Physiotherapy Evidence Database (PEDro)

Upper Extremity Lower extremity Physical Rehabilitation Motor signs motor symptoms Electromyographies Amyotrophic Lateral Sclerosis
